# A new, well-preserved genus and species of fossil Glaphyridae (Coleoptera, Scarabaeoidea) from the Mesozoic Yixian Formation of Inner Mongolia, China

**DOI:** 10.3897/zookeys.241.3262

**Published:** 2012-11-14

**Authors:** Zhuo Yan, Georgiy V. Nikolajev, Dong Ren

**Affiliations:** 1 Key Laboratory of Insect Evolution and Environmental Changes, College of Life Sciences, Capital Normal University, Beijing 100048, P R. China; 2 Al-Farabi Kazakh National University (Dept. of Biology and Biotechnology), al-Farabi Prospekt, 71, Almaty 050038, Kazakhstan

**Keywords:** Scarabaeoidea, Glaphyridae, new genus, new species, fossil, Early Cretaceous, Yixian Formation, Inner Mongolia, China

## Abstract

A new genus and species of fossil Glaphyridae, *Cretohypna cristata* **gen. et sp. n.**, is described and illustrated from the Mesozoic Yixian Formation. This new genus is characterized by the large body; large and strong mandibles; short labrum; elytra without longitudinal carina; and male meso- and possible metatibia apically modified. A list of described fossil glaphyrids of the world is provided. This significant finding broadens the known diversity of Glaphyridae in the Mesozoic China.

## Introduction

The family Glaphyridae MacLeay, 1819, is a relatively small group of Scarabaeoidea, currently comprising about 200 species and subspecies in four extant, two extant and fossil, and two fossil genera (Nikolajev et al. 2011). The fossil species of the Glaphyridae are placed into four genera. Two species of the extant genus *Glaphyrus* Latreille, 1807, have been found in the Miocene (Heer 1862; Krell 2007) and Early Cretaceous (Nikolajev and Ren 2011). One species of the extant genus *Lichnanthe* Burmeister, 1844 has been found in the Late Eocene (Carlson 2002). Two Mesozoic genera *Cretoglaphyrus* Nikolajev, 2005 and *Lithohypna* Nikolajev, Wang & Zhang, 2011 have been described from the Early Cretaceous (Nikolajev 2005; Nikolajev et al. 2011; Nikolajev and Ren 2012). A list of described fossil Glaphyridae of the world is summarized in the Table 1.

**Figure 1.**
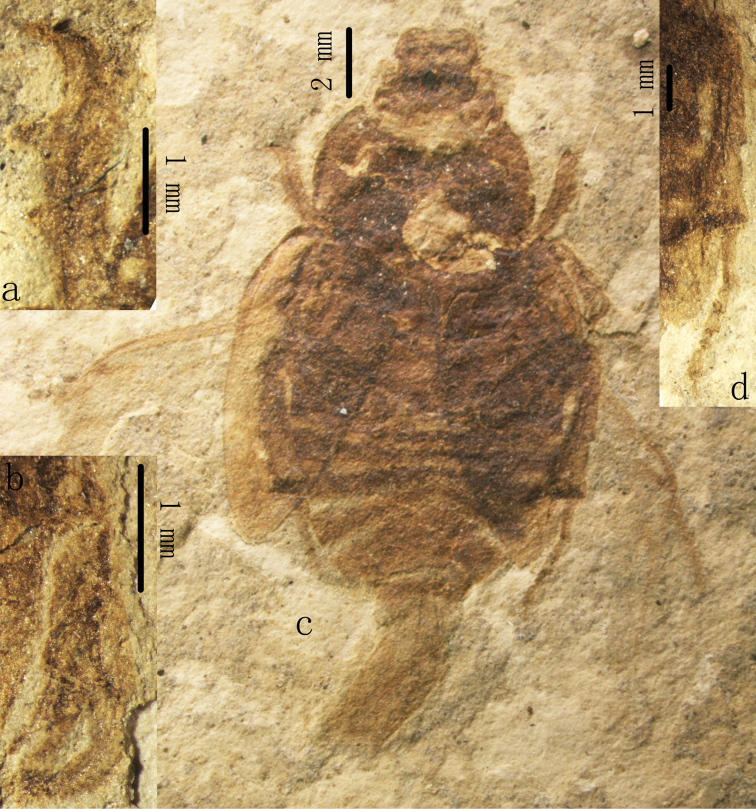
*Cretohypna cristata* Yan, Nikolajev & Ren gen. et sp. n., holotype, registration No. CNU-COL-NN2011003, **a** protibia **b** mesotibia **c** body in dorsal view **d** metatibia and metatarsus.

Recently, we collected an almost complete and well-preserved fossil specimen (Fig. 1) from the Yixian Formation near Liutiaogou Village, Ningcheng County, Chifeng City, Inner Mongolia, China. Based on a combination of its unique morphological characters (labrum 1.5 times shorter than in all described Glaphyridae, first segment of metatarsus 2.5 times longer than in all described fossil Glaphyridae, and lamellate apex of mesotibia), we erect a new genus and species, *Cretohypna cristata* Yan, Nikolajev & Ren gen. et sp. n. This species is placed in the family Glaphyridae based on the following characters: mandibles produced beyond apex of clypeus, eyes partially divided by canthus, abdomen with six visible sternites, pygidium visible beyond elytra, protibia with three large teeth on outer margin, and mesocoxae moderately separated. This significant finding, the first glaphyrid fossil species with preserved hind wings, broadens the known diversity of Glaphyridae in the Mesozoic China.

**Table 1.** Described fossil Glaphyridae (Coleoptera: Scarabaeoidea) of the world.

number	Species	Age	Locality
1	*Glaphyrus ancestralis* Nikolajev & Ren, 2011	Early Cretaceous	China
2	*Glaphyrus antiquus* Heer, 1862	Miocene	Germany
3	*Cretoglaphyrus calvescens* Nikolajev, 2005	Early Cretaceous	Russia
4	*Cretoglaphyrus leptopterus* Nikolajev, 2005	Early Cretaceous	Russia
5	*Cretoglaphyrus olenguicus* Nikolajev, 2005	Early Cretaceous	Russia
6	*Cretoglaphyrus rohdendorfi* Nikolajev, 2005	Early Cretaceous	Russia
7	*Cretoglaphyrus transbaikalicus* Nikolajev, 2005	Early Cretaceous	Russia
8	*Cretoglaphyrus zherikhini* Nikolajev, 2005	Early Cretaceous	Russia
9	*Lithohypna chifengensis* Nikolajev, Wang & Zhang, 2011	Early Cretaceous	China
10	*Lithohypna lepticephala* Nikolajev & Ren, 2012	Early Cretaceous	China
11	*Lithohypna longula* Nikolajev & Ren, 2012	Early Cretaceous	China
12	*Lithohypna tuberculata* Nikolajev & Ren, 2012	Early Cretaceous	China
13	*Lithohypna yuxiana* Nikolajev & Ren, 2012	Early Cretaceous	China
14	*Lichnanthe defuncta* (Wickham, 1910)	Late Eocene	America

## Material and methods

The study is based on one specimen collected near Liutiaogou Village, Ningcheng County, Chifeng City, from Yixian Formation of Inner Mongolia, China. The holotype is deposited in the Key Laboratory of Insect Evolution & Environmental Changes, College of Life Sciences, Capital Normal University, Beijing, China.

The specimen was examined with a Leica MZ12.5 stereomicroscope and illustrated with the aid of a drawing tube attached to the microscope. Line drawings were made using CorelDRAW X4 and Adobe Photoshop CS 5 graphic software.

Body length was measured along the midline from the mandibular apex to the apex of the pygidium and width was measured across the broadest part of abdomen. The length of pronotum was measured along the midline and the width was measured across the broadest part at its posterior angles. Abdomen length was measured along the midline and the width was measured across the broadest part.

The age of the Yixian Formation is debated. Three opinions have been proposed: Late Jurassic, Late Jurassic-Early Cretaceous and Early Cretaceous (Ren et al. 1997; Zheng et al. 2003; Cao 1999; Jin 1999; Chen et al. 2004; Wang et al. 2004; Wang et al. 2005; Swisher et al. 1999; Li et al. 2001; Zhou et al. 2003). Recent studies have confirmed that the Yixian Formation is the Early Cretaceous in age. The precise age is most likely restricted to 129.7-122.1 Ma (Barremian to early Aptian) (Chang et al. 2009; Yang et al. 2007; Zhou and Wang 2010; Zhu et al. 2007). The strata of Yixian Formation are mainly of lacustrine sediments intercalated with volcaniclastics (Ren et al. 1995). The stratigraphy and depositional environments of this area have been discussed in detail by Jiang and Sha (2007) and Jiang et al. (2011). The palaeoclimate of this area was recently interpreted as cool temperate with mean air temperatures of 10 ± 4 °C. (Amiot et al. 2011). The Yixian Formation has provided abundant fossil insects (Bai et al. 2010; Bai et al. 2011; Bai et al. 2012a; Bai et al. 2012b; Chang and Ren 2008; Liu et al. 2008; Yan et al. 2012a; Yan et al. 2012b; Yao et al. 2008; Zhang et al. 2010).

## Systematic paleontology

### Order Coleoptera Linnaeus, 1758. Superfamily Scarabaeoidea Latreille, 1802. Family Glaphyridae Macleay, 1819

#### Genus Cretohypna

Yan, Nikolajev & Ren gen. n.

urn:lsid:zoobank.org:act:687EC908-F0F6-425A-9604-72A9298F9C3F

http://species-id.net/wiki/Cretohypna

Figs 1 , 2

##### Type species.

*Cretohypna cristata* sp. n.

##### Etymology.

The generic name refers to the Cretaceous Period of its origin, and the generic name *Anthypna* Eschoscholtz, 1818. Gender: feminine.

##### Diagnosis.

Largeelongate oval and compact (head, pronotum and mesothorax are very close to each other) scarab beetle (Fig. 1c). Mandibles and labrum exposed beyond apex of clypeus and clearly visible in dorsal view of head, labrum approximately five times as wide as long. Pronotum subquadrate shaped with concave anterior margin and slightly convex lateral and posterior margins. Scutellum triangular. Mesoepimeron clearly visible from above between pronotum and elytron. Elytra convex and thin, without longitudinal carina; hind wings well-developed. Legs short and strong, mesocoxae moderately separated, protibia with three large teeth on outer margin (Fig. 1a), apex of male mesotibia lamellate (Fig. 1b, arrow); mesotibia and metatibia with 2 apical spurs; male metatarsus shorter than corresponding tibia (Fig. 1d). Abdomen with six visible sternites, first sternites not obscured by hind coxae. Pygidium exposed beyond apices of elytra.

##### Species composition.

Only the type species is known.

##### Distribution.

The genus is only known from the Early Cretaceous Yixian Formation, Liutiaogou Village, Ningcheng County, Chifeng City, Inner Mongolia, China.

##### Comparison.

According to the fossil record in the family Glaphyridae, there are three genera described from the Mesozoic Era: *Glaphyrus* (Nikolajev & Ren, 2011); *Cretoglaphyrus* (Nikolajev 2005) and *Lithohypna* (Nikolajev et al. 2011).

The new genus is readily distinguished from all Mesozoic Glaphyridae genera by very long first segment of male metatarsus. The new genus is distinguished from the genus *Glaphyrus* by lamellate apex of mesotibia and slender metafemur; from the genus *Cretoglaphyrus* by moderately separated mesocoxae and elytra without longitudinal carina; from the genus *Lithohypna* by short labrum and triangular scutellum.

#### Cretohypna cristata

Yan, Nikolajev & Ren sp. n.

urn:lsid:zoobank.org:act:AFA8B06D-B67F-4767-91F2-DA85652A625E

http://species-id.net/wiki/Cretohypna_cristata

Figs 1 , 2

##### Etymology. 

The specific name is derived from Latin word ‘*crista-’*, which means ‘comb’ or ‘crest’, refers to the presence of a transverse carina on the head.

##### Material.

Holotype, a well-preserved male specimen in dorsal view, registration No. CNU-COL-NN2011003. Housed in the Key Lab of Insect Evolution & Environmental Changes, College of Life Sciences, Capital Normal University, Beijing, China.

##### Diagnosis.

Same as the genus.

##### Description.

Body large, elongate oval and compact (Fig. 1c). Head nearly as wide as long, the widest part of head at its middle part, obviously narrower than pronotum, with transverse carina; mandibles strong, labrum and mandibles prominent, labrum exposed beyond apex of clypeus, labrum approximately five times as wide as long; mandibles exposed beyond apex of clypeus; eyes large and developed; anterior margin of clypeus moderately rounded, the presence of a transverse carina on the head. Pronotum transverse, nearly subquadrate; the widest part of pronotum at its base; anterior margin of pronotum concave; with lateral margins slightly convex, posterior margin slightly protruding. Scutellum small, triangular, about 2.2 times wider than long. Elytra long and narrow, slightly constricted to the basis, with lateral margins slightly convex, without longitudinal carina, with weak striae on lateral part, convexly constricted to the apex in the apical quarter, dehiscent. Hind wing: both hind wings preserved, well-developed, the RP, RA3+4, RA_4_+RP_1_ veins preserved on the fossil. Abdomen with six visible sternites, the first sternites not obscured by hind coxae, pygidium exposed beyond apices of elytra. Genitalia preserved and curved. Procoxa about 2.3 times wider than long, mesocoxae moderately separated. Meso- and metafemur slender. Protibia with three large teeth on outer margin (Fig. 1a); mesotibia possibly with two transverse carinae on outer margin (Fig. 1b); two spurs at the end of meso- and metatibia; spurs of mesotibia differing distinctly in length; spurs of metatibia subequal in length. Only two mesotarsomeres are preserved in this specimen, relative length of each segment (base to apex) 59: 49; metatarsus with five segments, relative length of each segment (base to apex) 120: 45: 45: 45: 68 (Fig. 1d).

**Figure 2.**
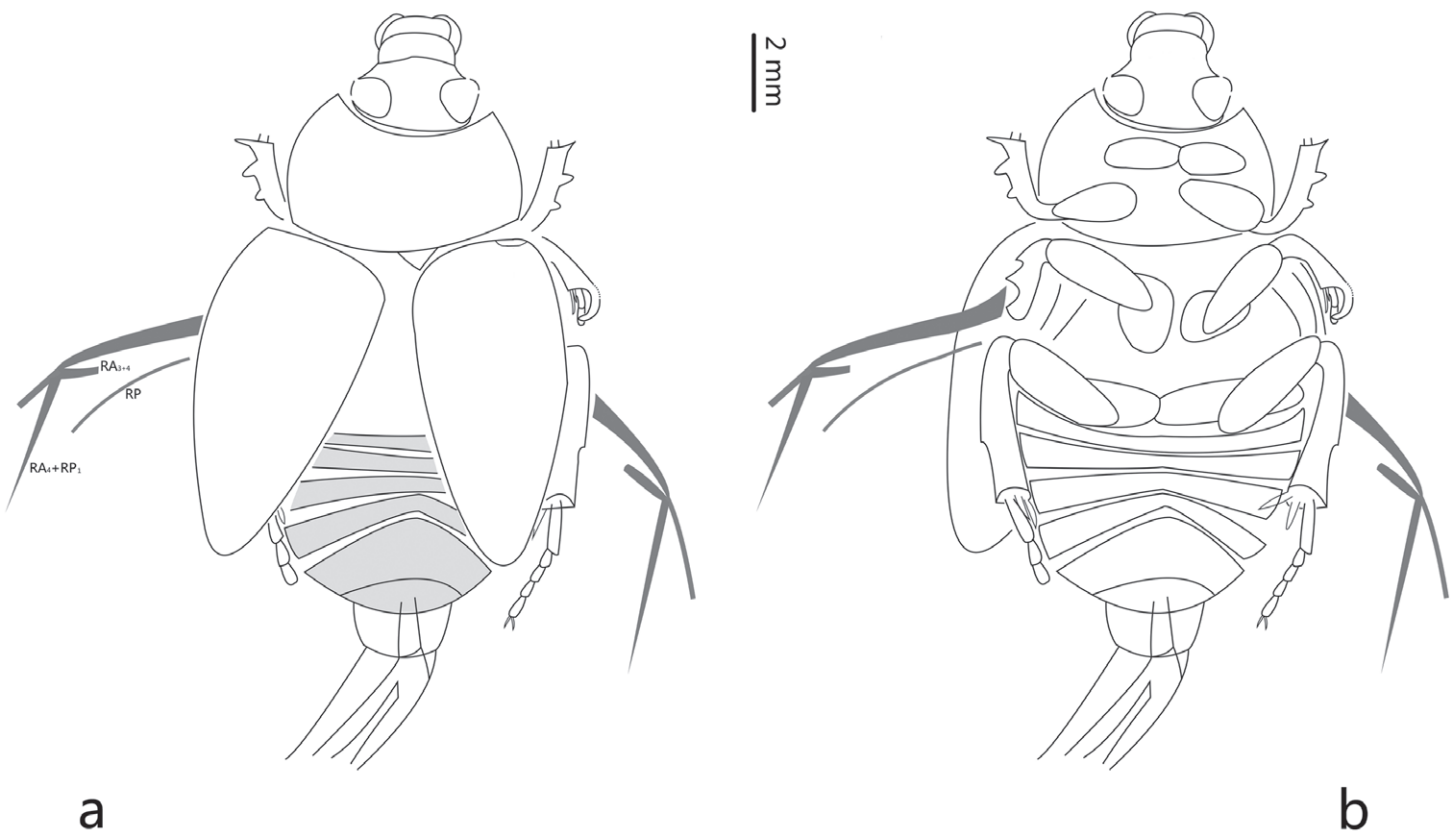
*Cretohypna cristata* Yan, Nikolajev & Ren gen. et sp. n., line drawings of holotype **a** dorsal view **b** ventral view.

##### Measurements. 

Body length 16.1 mm, greatest body width 8.4 mm, head length 3.0 mm, head width 3.4 mm, pronotum length 3.1 mm, pronotum width 6.0 mm, elytron length 8.7 mm, elytron width 4.2 mm, length of abdominal segments: 1–0.4 mm, 2–0.3 mm, 3–0.5 mm, 4–0.4 mm, 5–1.5 mm, 6 –0.9 mm.

## Supplementary Material

XML Treatment for Cretohypna

XML Treatment for Cretohypna cristata
